# Supplementary material for: Comparative Analysis of the Gut Microbiota of Bat Species with Different Feeding Habits
Source: Biology (Basel). 2024 May 22;13(6):363. doi: 10.3390/biology13060363 (PMC11200740; doi:10.3390/biology13060363)
Supplement: Supplementary file 1 [file biology-13-00363-s001.zip › Table S1.docx]

|  |  |  | |  |
| --- | --- | --- | --- | --- |
| **Sample ID** | **Reads Before Filtering** | **Reads After Filtering** | **Goods Coverage** | |
| N01 | 54841 | 33642 | 99.97 | |
| N02 | 40971 | 19630 | 99.95 | |
| N03 | 48243 | 29517 | 99.94 | |
| N04 | 54977 | 29712 | 99.99 | |
| N05 | 46548 | 24941 | 99.94 | |
| N06 | 54253 | 32189 | 99.98 | |
| N07 | 46364 | 22060 | 99.98 | |
| F01 | 212243 | 146247 | 99.84 | |
| F02 | 227481 | 143625 | 99.77 | |
| F03 | 184071 | 108519 | 99.89 | |
| F04 | 254787 | 166054 | 99.91 | |
| F05 | 237661 | 182597 | 99.95 | |
| F06 | 199860 | 157957 | 99.94 | |
| F07 | 152330 | 105378 | 99.96 | |
| F08 | 158076 | 114968 | 99.91 | |
| F09 | 171170 | 130552 | 99.91 | |
| F10 | 115952 | 88437 | 99.90 | |
| I01 | 118696 | 82353 | 99.92 | |
| I02 | 178686 | 127457 | 99.91 | |
| I03 | 172701 | 117838 | 99.90 | |
| I04 | 198335 | 140711 | 99.89 | |
| I05 | 210785 | 149087 | 99.95 | |
| I06 | 189711 | 130934 | 99.81 | |
| I07 | 221179 | 149622 | 99.92 | |
| I08 | 212413 | 161160 | 99.91 | |
| I09 | 130446 | 98119 | 99.93 | |
| I10 | 120349 | 80453 | 99.77 | |
| H01 | 124068 | 94806 | 99.86 | |
| H02 | 130188 | 97056 | 99.92 | |
| H03 | 169790 | 123759 | 99.94 | |
| H04 | 147658 | 109439 | 99.81 | |
| H05 | 169966 | 125496 | 99.90 | |
| H06 | 165236 | 116060 | 99.95 | |
|  |  |  | |  |
