# Supplementary material for: Comparative Analysis of the Gut Microbiota of Bat Species with Different Feeding Habits
Source: Biology (Basel). 2024 May 22;13(6):363. doi: 10.3390/biology13060363 (PMC11200740; doi:10.3390/biology13060363)
Supplement: Supplementary file 1 [file biology-13-00363-s001.zip › Tables S3 and S4.docx]

**Table S3.** List of taxa at the genus level shared by all 4 bat species.

| **Genus** |
| --- |
| Mycoplasma Enterobacteriaceae_un Clostridium_sensu_stricto_1 Clostridiaceae_un Pasteurellaceae_un Streptococcus Neisseriaceae_un Bacilli_un Lactobacillales_un Lactobacillus Lactococcus Helicobacter Pseudomonas Micrococcaceae_un Acinetobacter Staphylococcus Comamonadaceae_un Lactobacillaceae_un Lachnospiraceae_un Corynebacterium Dietzia Bacteroides Lawsonella Limosilactobacillus Faecalibacterium Enterobacterales_un Ligilactobacillus Ruminococcaceae_un Oscillospiraceae_un Peptostreptococcaceae_un Rhizobiaceae_un Chryseobacterium Butyricicoccus Erysipelatoclostridium Bacillales_un Hafnia_Obesumbacterium Alistipes Brevibacterium Shewanella Parabacteroides Sphingomonadaceae_un Stenotrophomonas UCG_005 Brachybacterium Romboutsia |

**Table S4.** List of taxa at the genus level unique to a group.

| Insectivorous | Hematophagous | Frugivorous | Nectarivorous |
| --- | --- | --- | --- |
| Murdochiella Arcanobacterium Actinomycetaceae_un Corynebacteriaceae_un Weeksellaceae_un Mageeibacillus Zoogloea Dysgonomonas Cardiobacteriaceae_un Campylobacterales_un Family_XI_un Leptotrichiaceae_un Oscillibacter Proteocatella Saccharimonadaceae_un CAG_873 Prevotella NK4A214_group Sulfuricurvum UCG_002 Burkholderiales_un M2PB4_65_termite_group_ge Nosocomiicoccus Trichlorobacter Aminomonas Desulfobulbaceae_un Geobacteraceae_un Ruminococcus Tolumonas Gastranaerophilales_ge Prevotellaceae_NK3B31_group Anaerolineaceae_UCG_001 Ferribacterium Geobacter Oxalobacteraceae_un Selenomonadaceae_un Brachymonas Flavobacterium Lachnospiraceae_UCG_010 Oscillospirales_un Propionivibrio Rhodopirellula | Pasteurella WPS_2_ge Gemmataceae_un Bergeyella Unknown_Family_ge Ktedonobacteraceae_un Tomitella Pygmaiobacter Corynebacteriales_un Sphingomonas Aminobacter Crossiella alphaI_cluster Aquisphaera DSSD61 Nocardia Christensenellaceae_R_7_group Globicatella Marvinbryantia Mogibacterium Erysipelotrichaceae_ge Rhizobiales_un | Chlamydiales_un Pir4_lineage JG30_KF_CM45_ge Luteimonas Aeromicrobium Intrasporangiaceae_un Micromonosporaceae_un Moraxellaceae_un Rhodobacteraceae_un Pedomicrobium Actinotignum | Ureaplasma Alysiella Wolbachia Bartonella Paraclostridium Capnocytophaga Leptotrichia Leuconostoc Actinobacillus Cardiobacterium Nocardiaceae_un |
