# Supplementary figures and images for: Comparative Analysis of the Gut Microbiota of Bat Species with Different Feeding Habits
Source: Biology (Basel). 2024 May 22;13(6):363. doi: 10.3390/biology13060363 (PMC11200740; doi:10.3390/biology13060363)

## Slide 1
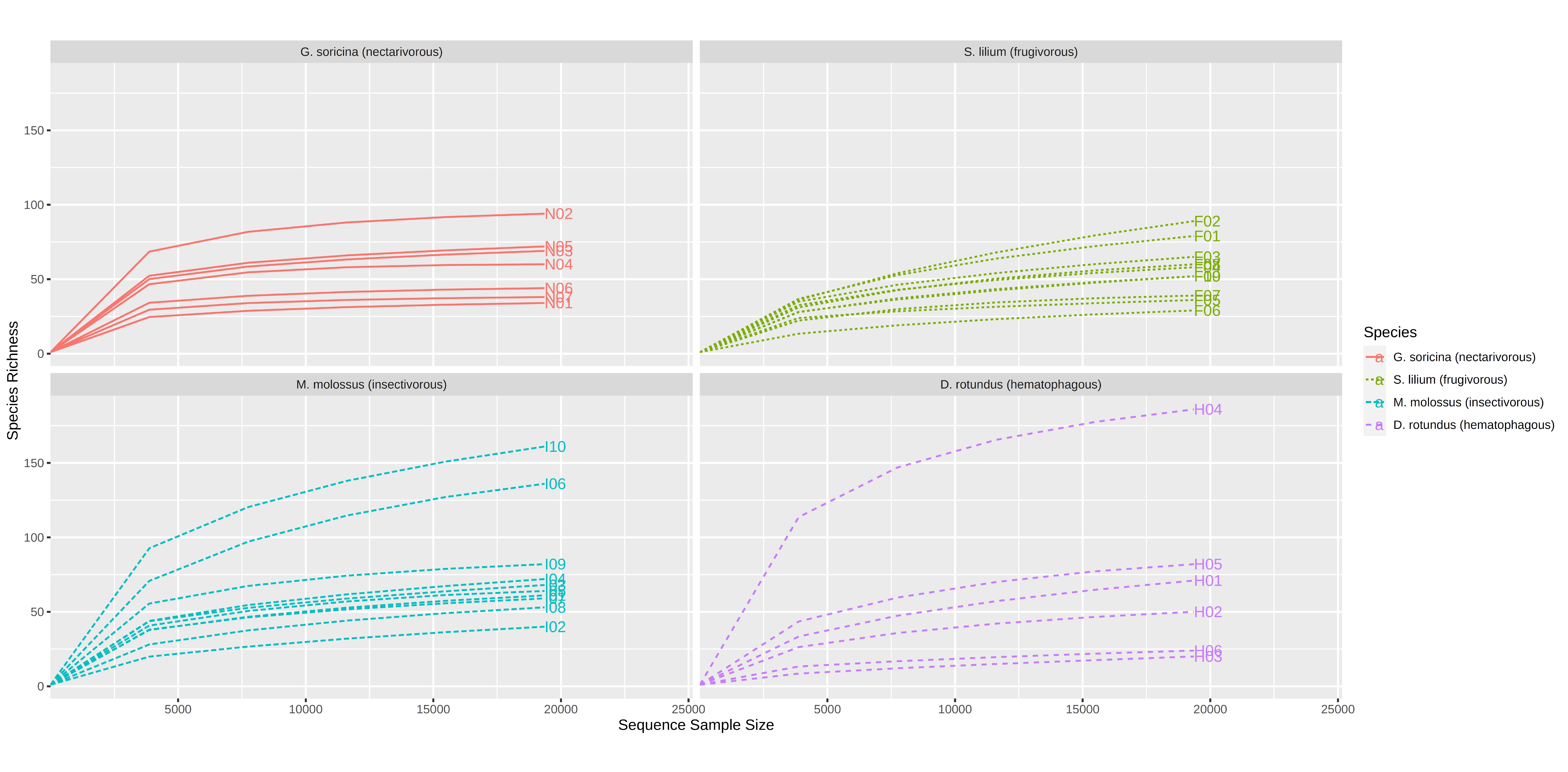

Supplement: Supplementary file 1 [file biology-13-00363-s001.zip › Fig. S1.pptx]

## Slide 1
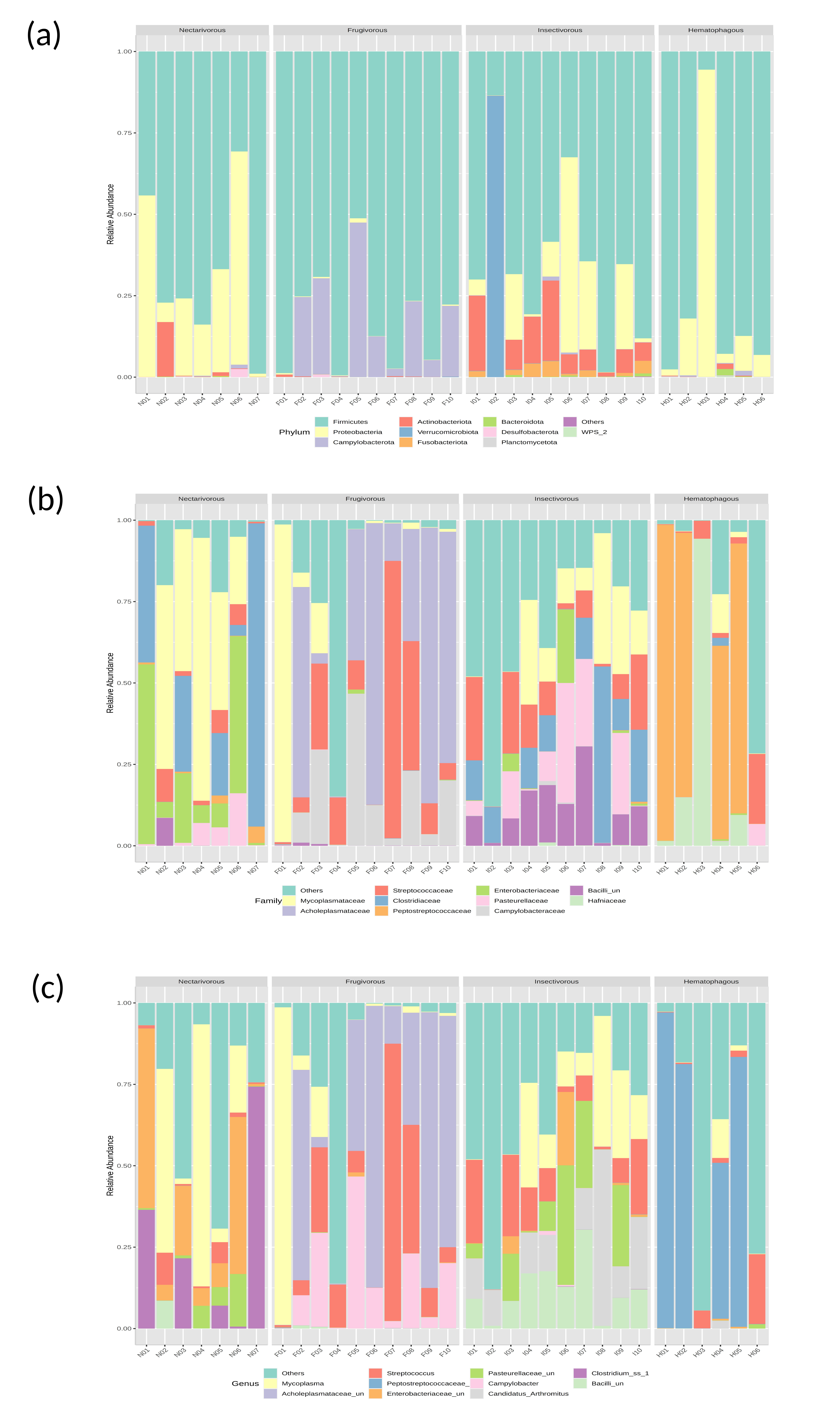

(a)
(b)
(c)

Supplement: Supplementary file 1 [file biology-13-00363-s001.zip › Fig. S2.pptx]
